# Facial Edema Induced by Glycopyrrolate in a Patient With Metastatic Tongue Cancer: A Case Report

**DOI:** 10.7759/cureus.69024

**Published:** 2024-09-09

**Authors:** Abrar Khojah, Shumukh Alqahtani, Zaid A Majeed, Faisal B Almatrafi, Wafaeiy Shiqdar

**Affiliations:** 1 Palliative Care, Soliman Al-Habib Hospital, Jeddah, SAU; 2 Department of Medicine and Surgery, College of Medicine, Umm Al-Qura University, Makkah, SAU; 3 Recovery, National Guard Health Affairs, Jeddah, SAU

**Keywords:** drug side effect, glycopyrrolate, palliative cancer, rare case report, tongue cancer

## Abstract

Glycopyrrolate, an anticholinergic medication, is commonly used for managing excessive secretions in palliative care, especially in patients with advanced head and neck cancers. However, its side effect profile, particularly in complex oncological cases, is not fully understood. This case report presents a 62-year-old male with metastatic squamous cell carcinoma (SCC) of the tongue, who was managed in a palliative care setting. Glycopyrrolate 0.2 mg subcutaneously (SC) or intravenously (IV) every six hours was initiated to control chest secretions. While the patient showed initial symptomatic improvement, he subsequently developed significant and unexpected facial edema extending to both lower eyelids. The edema did not respond to antibiotics or corticosteroids and only improved after discontinuation of glycopyrrolate, suggesting a potential adverse reaction, although the advanced stage of his illness may have also contributed to the development of edema. This case underscores the importance of monitoring for rare adverse effects like facial edema when using glycopyrrolate, particularly in patients with advanced metastatic cancers. Further research is warranted to explore the underlying mechanisms of this reaction and guide safer clinical practice.

## Introduction

Tongue cancer, though relatively rare, constitutes about 1% of all new cancer cases annually in the United States [1]. It is more commonly diagnosed in men, with an incidence rate of 3.5 per 100,000 individuals per year, typically between the ages of 55 and 64 [1]. The prognosis for tongue cancer varies significantly depending on the stage at diagnosis, with a five-year relative survival rate of 82.1% if localized to the primary site, dropping to 39.8% if metastasized [2].

The most prevalent form of tongue cancer is squamous cell carcinoma (SCC), which is categorized based on the tumor’s location: SCC of the oral tongue (anterior two-thirds) and SCC of the oropharynx (posterior one-third) [3]. Common risk factors include cigarette smoking, alcohol consumption, human papillomavirus (HPV) infection, genetic predispositions, and poor oral hygiene [4].

In the context of palliative care for advanced tongue cancer, managing symptoms such as excessive chest secretions is crucial for improving the patient's quality of life [5,6]. Anticholinergic medications like glycopyrrolate are often employed to reduce salivary and respiratory secretions, given their effectiveness and relatively low central nervous system side effects due to limited blood-brain barrier penetration [7-9].

A few studies have explored the use of glycopyrrolate in managing secretions in oncological patients. For example, in patients with esophageal cancer, glycopyrrolate has been reported to effectively reduce secretions with minimal side effects [8]. Similarly, management strategies for salivary flow in head and neck cancer patients have occasionally included glycopyrrolate, though detailed reports on its safety profile in this population are limited [10]. Given the limited data available, our case report provides a valuable addition to the existing literature by highlighting a rare but significant adverse effect of glycopyrrolate, facial edema, in a patient with metastatic SCC of the tongue. This case underscores the need for careful monitoring when using glycopyrrolate in complex oncological cases, where multiple factors may influence drug reactions. Further research is warranted to better understand the side effect profile of glycopyrrolate in this specific patient population and to guide safer clinical practice.

The purpose of this report is to add to the existing literature on the side effects of glycopyrrolate in palliative care, emphasizing the importance of careful monitoring when prescribing this medication to patients with advanced cancer. This case is particularly significant because it identifies facial edema as a potential side effect of glycopyrrolate, a finding that could have important implications for future clinical practice and patient safety.

## Case presentation

A 62-year-old male with a history of advanced metastatic SCC of the tongue, which had spread to the cervical lymph nodes and lungs, was admitted due to a malignant wound infection and increased chest secretions. His medical history was significant for hypertension, type 2 diabetes mellitus, and chronic obstructive pulmonary disease (COPD), all of which were managed with appropriate medications. The patient had experienced aspiration while drinking soup the day before admission, leading to a worsening of his chest secretions. He also reported neck pain, described as pressure-like, which was effectively managed with tramadol.

Upon examination, the patient appeared stable with a palliative performance scale (PPS) of 50%, indicating moderate disability. He was afebrile, had a tracheostomy in place, and was able to communicate through writing. The patient noticed that fluids occasionally leaked through a wound in his neck, although his cough and secretions showed some improvement. He experienced intermittent nausea, which was controlled with metoclopramide, and developed constipation on the first day of admission, for which suppositories were administered. Table 1 summarizes the patient's clinical course, including the key interventions and outcomes.

**Table 1:** Treatment timeline for management of a case of metastatic tongue cancer with glycopyrrolate and subsequent complications SCC: squamous cell carcinoma; PPS: palliative performance scale; SC: subcutaneous; IV: intravenous; Q6h: every six hours; OD: once daily

Day	Event	Details/outcome
Day 0	Admission and initial symptoms	Patient admitted with metastatic SCC of the tongue, malignant wound infection, increased chest secretions, and neck pain managed with tramadol.
Day 1	Examination and initial management	PPS of 50%, stable condition, tracheostomy in place, started on glycopyrrolate 0.2 mg SC/IV Q6h for secretion management; hypertonic saline stopped, steroid nebulizer continued.
Day 2	Monitoring and symptom management	Cough and secretions improved; nausea controlled with metoclopramide; senna prescribed for constipation.
Day 3	Surgical consultation	Evaluation of fluid leakage from neck wound during drinking; suspected cutaneous esophageal fistula deemed non-surgical due to malignancy.
Day 4-5	Continued management	Chest secretions thickened, suctioning became difficult; glycopyrrolate continued; pain and cough managed effectively.
Day 7-8	Development of facial edema	Significant facial edema developed, initially treated with antibiotics for suspected abscess; dexamethasone 4 mg OD started with brief symptom relief.
Day 9-13	Dexamethasone trial and outcome	Brief improvement with dexamethasone, but edema continued to worsen, leading to discontinuation of dexamethasone.
Day 14	Discontinuation of glycopyrrolate	Gradual improvement in edema was observed afterward.
Day 20	Outcome and complications	The patient died after developing fever and diarrhea, later succumbing to septic shock from a Salmonella infection.

Further examination revealed a malignant wound on both sides of the patient’s neck, with concerns about a possible oral fistula forming from the wound to the skin. To manage his upper airway secretions and cough, hypertonic saline was discontinued, a steroid nebulizer was continued, and due to the patient's significant dysphagia and the need for rapid control of chest secretions, glycopyrrolate 0.2 mg was administered subcutaneously (SC) or intravenously (IV) every six hours. The tracheostomy team was involved in follow-up care, and chest physiotherapy was started. The next day, the patient’s cough and secretions were reduced, though he continued to refuse suppositories, so Senna was prescribed for constipation.

The patient was advised on safe eating techniques, particularly avoiding the left side of his mouth where the malignant wound was located. Surgical consultation was obtained due to the complexity of his condition, which included a fungating malignant wound in the neck. Despite these interventions, the patient continued to experience fluid leaks from the neck wound while drinking. The general surgery team diagnosed a cutaneous esophageal fistula, which was deemed unlikely to heal due to the extent of the malignancy, ruling out surgical intervention.

Over the following days, the patient’s chest secretions thickened, making suctioning difficult, although there were no further complaints of fluid leakage. Pain and cough were well managed, but glycopyrrolate remained necessary to control chest secretions. A week later, the patient developed significant facial edema, initially suspected to be an abscess, and treated with antibiotics. When the edema did not improve, dexamethasone 4 mg OD was administered, leading to brief symptom relief. However, symptoms soon worsened, and dexamethasone was discontinued.

**Figure 1:**
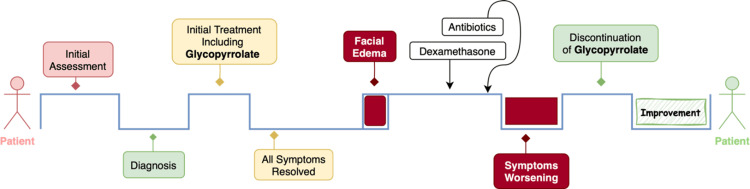
Timeline of facial edema following glycopyrrolate use in tongue cancer patient

The patient’s facial edema, extending to both lower eyelids, persisted and worsened over the next few days. Glycopyrrolate was suspected as the cause of the edema and was discontinued, leading to gradual improvement and stabilization of the patient’s condition. Figure 1 shows the timeline of facial edema following glycopyrrolate. However, the patient subsequently developed fever and diarrhea and eventually succumbed to septic shock due to a Salmonella infection.

This case illustrates the complex challenges in managing advanced tongue cancer, including the unexpected development of facial edema as a side effect of glycopyrrolate, highlighting the need for careful monitoring and treatment adjustments.

## Discussion

Patients with tongue cancer frequently endure a variety of distressing symptoms, including pain and dysphagia, which significantly impact their quality of life. While drooling is not commonly reported in these patients, it can be particularly distressing when it occurs [11]. Dysphagia, along with loss of sensitivity and deformities in the oral cavity and tongue due to tumor growth or anticancer treatments, often leads to altered saliva production, ranging from excessive sialorrhea to reduced secretion [12]. In the patient discussed in this case report, drooling and excessive respiratory tract secretions were the primary symptoms that necessitated management. Despite being frequently overlooked, effective mouth care remains a critical component of palliative care, as the associated oral complications can cause significant discomfort throughout all stages of the disease [13].

Anticholinergic medications like glycopyrrolate are commonly used in palliative care settings to manage symptoms such as bowel obstruction and death rattle by reducing gastrointestinal and bronchial secretions [14]. However, there is limited evidence supporting their efficacy in managing excessive salivation (sialorrhea) in cancer patients, particularly in those with head and neck cancers like tongue cancer [15]. In most cases, sialorrhea in tongue cancer patients is uncommon, and the literature on the subject is sparse [9].

Glycopyrrolate, a quaternary ammonium anticholinergic, is frequently administered preoperatively to reduce salivation and respiratory secretions [16]. It is reported to be more effective than atropine in inhibiting salivary secretion, with a selective and sustained effect [8]. Additionally, glycopyrrolate is associated with minimal central nervous system (CNS) side effects due to its poor penetration of the blood-brain barrier [17]. These properties make it a favorable choice in managing conditions associated with excessive secretions.

While the use of glycopyrrolate in tongue cancer patients is limited in the literature, a few studies have reported its effectiveness in managing sialorrhea and drooling. These studies generally suggest that glycopyrrolate may be beneficial, but the data remains sparse, particularly concerning its use in patients with head and neck cancers [9]. More research is needed to explore the full range of its efficacy and safety in this specific patient population, especially given the potential for rare side effects like facial edema.

The case presented in this report adds a unique perspective to the existing body of knowledge by highlighting an unexpected and significant side effect, facial edema, following the administration of glycopyrrolate. The onset of facial edema, particularly affecting the bilateral lower eyelids, is an uncommon reaction not extensively reported in the literature. While glycopyrrolate is generally well-tolerated, especially given its limited CNS effects, its potential to cause facial edema presents a notable exception to its otherwise favorable safety profile [16,18].

Previous studies have reported various side effects of glycopyrrolate, including behavioral irritability, urinary retention, and constipation, particularly in pediatric populations or in patients with preexisting conditions such as prostatic hypertrophy [16,19]. For example, a study focusing on esophageal cancer patients highlighted the occurrence of urinary retention in a patient with prostatic hypertrophy, attributed to the smooth muscle relaxation effects of glycopyrrolate [20]. This example illustrates one of the drug’s known side effects, particularly in patients with preexisting conditions like prostatic hypertrophy [19]. The study emphasizes the importance of considering the drug's potential impact on the urinary tract when administered to cancer patients, as it can lead to complications such as decreased motility and urinary retention. Furthermore, studies involving children with excessive sialorrhea who were treated with glycopyrrolate reported side effects such as constipation, facial flushing, and nasal congestion, but facial edema was not commonly noted [10].

Considering this prior report, the facial edema observed in our patient prompts important questions about the underlying mechanisms involved. The rarity of this side effect in the literature highlights the necessity for clinicians to exercise caution when administering glycopyrrolate, especially in vulnerable populations such as patients with advanced cancer.

The development of facial edema in this patient could be attributed to several potential mechanisms, none of which are entirely conclusive but all of which warrant further investigation. One possible explanation is that glycopyrrolate, while not significantly crossing the blood-brain barrier, may still exert peripheral anticholinergic effects that lead to fluid retention and subsequent edema [17]. Anticholinergic drugs reduce secretions and can lead to decreased lymphatic drainage, which might explain the accumulation of fluid in the facial tissues [16].

Another hypothesis could involve an immune-mediated response, where glycopyrrolate triggers an inflammatory reaction in certain individuals, leading to localized edema [18]. This immune response could be related to the patient’s underlying condition, advanced metastatic cancer, where the immune system is already compromised and could react unpredictably to medications [2]. However, this is purely speculative and would require detailed immunological studies to confirm.

A third possibility is that the facial edema was not solely due to glycopyrrolate but was a result of a cumulative effect of the medication combined with the patient’s existing conditions, including the presence of a malignant wound, tracheostomy, and possible lymphatic obstruction caused by the tumor spread [21]. In this scenario, glycopyrrolate might have exacerbated a preexisting tendency toward fluid retention and edema, particularly in the facial region where lymphatic drainage could already be compromised by the tumor’s spread to cervical lymph nodes.

Additionally, this case emphasizes the need for further research into the use of glycopyrrolate in cancer patients, particularly those with advanced disease. While glycopyrrolate is generally considered safe and effective, its use in this population is not well-studied, and the potential for rare but significant side effects should not be overlooked. Future studies should aim to better understand the mechanisms by which Glycopyrrolate may cause edema and identify patient populations that may be at higher risk for such side effects.

## Conclusions

This case highlights the critical importance of individualized care in managing patients with advanced cancer, particularly when using commonly prescribed medications like glycopyrrolate in palliative care settings. The unexpected development of facial edema in this patient underscores the need for clinicians to remain vigilant and to monitor closely for rare and unusual side effects. This case contributes to clinical practice by emphasizing that even well-established medications can have unpredictable effects in complex cases. It also calls for careful consideration and prompt reassessment of treatment plans when adverse reactions occur, while acknowledging the multifactorial nature of symptom development in advanced cancer patients.
